# Involvement of transcribed lncRNA uc.291 and SWI/SNF complex in cutaneous squamous cell carcinoma

**DOI:** 10.1007/s12672-021-00409-6

**Published:** 2021-05-03

**Authors:** M. Mancini, A. Cappello, R. Pecorari, A. M. Lena, M. Montanaro, L. Fania, F. Ricci, G. Di Lella, M. C. Piro, D. Abeni, E. Dellambra, A. Mauriello, G. Melino, E. Candi

**Affiliations:** 1 Istituto Dermopatico Dell’Immacolata-IRCCS, via dei Monti di Creta 104, 00167 Rome, Italy; 2 Department of Experimental Medicine, University of Rome “Tor Vergata”, via Montpellier 1, 00133 Rome, Italy

**Keywords:** Epidermis, Basal cell carcinoma, Squamous cell carcinoma, ACTL6A, SWI/SNF complex, LncRNA

## Abstract

While non-melanoma skin cancers (NMSCs) are the most common tumours in humans, only the sub-type cutaneous squamous cell carcinoma (cSCC), might become metastatic with high lethality. We have recently identified a regulatory pathway involving the lncRNA transcript uc.291 in controlling the expression of epidermal differentiation complex genes via the interaction with ACTL6A, a component of the chromatin remodelling complex SWI/SNF. Since transcribed ultra-conserved regions (T-UCRs) are expressed in normal tissues and are deregulated in tumorigenesis, here we hypothesize a potential role for dysregulation of this axis in cSCC, accounting for the de-differentiation process observed in aggressive poorly differentiated cutaneous carcinomas. We therefore analysed their expression patterns in human tumour biopsies at mRNA and protein levels. The results suggest that by altering chromatin accessibility of the epidermal differentiation complex genes, down-regulation of uc.291 and BRG1 expression contribute to the de-differentiation process seen in keratinocyte malignancy. This provides future direction for the identification of clinical biomarkers in cutaneous SCC. Analysis of publicly available data sets indicates that the above may also be a general feature for SCCs of different origins.

**Supplementary Information:**

The online version contains supplementary material available at 10.1007/s12672-021-00409-6.

## Introduction

The epidermis is a pluri-stratified and keratinized epithelium comprising a proliferative compartment (basal layer) and a multi-stage differentiation compartment (spinous, granular and corneous layers). The epidermis gives rise to two common cancer-types derived by the proliferating keratinocytes (basal cell carcinoma, BCC) and differentiating keratinocytes (cutaneous squamous cell carcinoma, cSCC). BCC, the most common type of NMSC, accounts for almost 90% of all skin cancers [1] and originates from the interfollicular epidermis stem cells, the hair follicle infundibulum cells or the hair follicle bulge cells [2–4]. BCCs develop following UV-induced mutagenesis [5]. Sequencing data indicate that 85% of mutations were in Shh signalling genes [2, 6]. cSCCs is the second most frequent type of skin malignancy [7]. It usually arises from the in situ lesions named actinic keratosis, or it may grow in photo-exposed areas as de novo lesion [7]. cSCCs develop from keratinocytes with multifactorial pathogenesis. They are driven by environmental carcinogens such us UV radiations in combination with abnormal immune surveillance, genetic and epigenetic alterations and genetic host susceptibility (i.e. oncogenic viruses and chronic inflammations) [8, 9]. Genomic analysis data revealed several oncogenic mutations in cSCCs leading to the dysregulation of several pathways, also underlying tumour heterogeneity [10–12]. Among them, mutations in p53, Notch1/2, TGF-beta receptor inactivation and p63 dysregulation are the most frequents genomic alterations [3, 13–21]. These data have potential prognostic and personalised medicine values [21–28].

Abnormalities of the SWI/SNF complex has been associated to a number of human malignancies, resulting in one of the most affected epigenetic modifiers in cancer. Around 20% of human tumors carries mutations, translocations and/or deletions involving different subunits of the SWI/SNF complex, including the catalytic subunits BRG1 and BRM [29, 30], making this complex as one of the most commonly affected targets in tumors [30, 31]. The SWI/SNF abnormalities found in cancer were loss-of-function mutations, leading to the idea of the SWI/SNF complex as a tumor suppressor complex [32].

In the last decade, long non-coding RNAs (lncRNAs) as well as miRNAs, have generated significant interest in controlling different stages of organismal development and diseases, including carcinomas formation [33, 34]. While the mutational background for cSCC and BCC is well documented, the role of lncRNAs as players in the complex cancer signaling network of skin malignancies remain poorly understood [35, 36]. So far, only few lncRNAs have been described to play a role in controlling cSCCs [37–39]. Among them, the oncogenic LINC00162 (also named PICSAR) and HOTAIR [29, 40–42] and the tumour suppressor LINC00520 [43].

We have previously reported that a specific sub-class of lncRNAs containing ultraconserved regions (T-UCRs) [44–46] are involved in controlling somatic stem cell fate and epithelial differentiation. Specifically, we demonstrated that the lncRNA containing ultraconserved region, uc.291, is implicated in chromatin remodelling to allow the expression of keratinocyte differentiation genes [47]. Uc.291 physically interacts with ACTL6A (BAF53a), following uc.291-ACTL6A interaction, the chromatin-remodelling complex SWI/SNF is able to interact with differentiation promoters and releases chromatin to allow the transcription of the epidermal differentiation complex (EDC) genes, including loricrin, filaggrin and LCE1B [47]. Interestingly, ACTL6A is also amplified at genomic level and highly expressed in head and neck squamous cell carcinomas (HNSCC) [48]. In HNSCC, ACTL6A modulates the SWI/SNF complex to suppress differentiation. Basically, ACTL6a prevents SWI/SNF complex binding to differentiation gene promoters [48]. This opens up an unexplored possibility that uc.291 could also play a role in head and neck and cutaneous squamous cell carcinomas formation.

Here, we explore the hypothesis that uc.291/ACTL6A and BRG1, the catalytic subunit of the SWI/SNF complex, are dysregulated in cSCCs leading to downregulation of keratinocyte differentiation programme, typical of poorly differentiated carcinomas. We evaluated uc.291/ACTL6A/BRG1 expression at mRNA level in a cohort of 39 human tumour specimens as well as the expression at protein level by tissue microarray (TMA). Results indicate that uc.291 and BRG1 are downregulated in cSCC and BCCs, as well as the expression of their target loricrin and LCE1C, indicating that the epigenetic modulations mediated by uc.291/SWI/SNF complex are involved in cSCC pathogenesis.

## Results

### BRG1 and uc.291 are downregulated in NMSCs

**Fig. 1:**
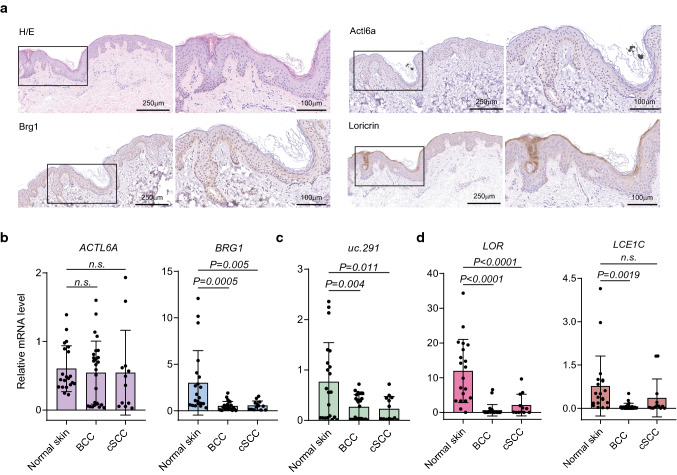
Expression of BRG1, ACTL6A, uc.291 and the differentiation genes loricrin and LCE1C in normal epidermis and NMSCs. **a** H&E staining and IHC analysis of Actl6a, Brg1 and Loricrin in normal skin. One representative image of 4 is shown. **b** *BRG1* and *ACTL6A* expression at the mRNA level. Relative quantification of clinical samples of normal skin (n = 21), cBCC (n = 26) and cSCC (n = 13) by RTqPCR. The p-value was obtained using one-way ANOVA; *ns* not significant. **c** *uc291* expression at the mRNA level. Relative quantification of clinical samples of normal skin (n = 21), cBCC (n = 26) and cSCC (n = 13) by RTqPCR. The p-value was obtained by one-way ANOVA; *ns* not significant. **d** *LORICRIN and LCE1C* expression at the mRNA level. Relative quantification of clinical samples of normal skin (n = 21), cBCC (n = 26) and cSCC (n = 13) by RTqPCR. The p-value was obtained by one-way ANOVA; *ns* not significant

The SWI/SNF complex has been linked to a number of human malignancies, resulting in one of the most affected targets in cancer [30, 49], however, its expression in cSCC and BCC has yet to be investigated. We collected and analysed 39 primary NMSCs (26 BCCs and 13 cSCCs). The clinicopathological features are detailed in Supplementary Table 1. The perilesional epidermis and normal skin were included as controls (n = 21). We first evaluated the expression of ACTL6A and BRG1 in the human skin epidermis (Fig. 1a). Immunohistochemistry revealed heterogeneous expression of BRG1 and ACTL6A in keratinocytes nuclei, with intense staining in the basal layer of the epidermis. We included haematoxylin–eosin staining and, and as control, the immunohistochemistry of the late differentiation marker loricrin (Fig. 1a). We then investigated BRG1 and ACTL6A expression in the collected primary tumours by RTqPCR (Supplementary Table 1). ACTL6A and BRG1 were expressed differently between BCCs/cSCCs and normal controls. Although BRG1 expression was strongly downregulated in both BCCs and cSCCs, ACTL6A expression was unchanged or tended to increase (Fig. 1b). Interestingly, uc.291 expression paralleled that of BRG1 and was strongly downregulated in both cancer types (Fig. 1c). As a consequence, we observed a strong reduction in the expression of loricrin and LCE1C, two genes located in the EDC locus, both positively regulated by uc.291 and BRG1 [47]. These data indicate that in BCCs and cSCCs, BRG1 and uc.291 downregulation accounts for ACTL6A-mediated suppression of the expression of differentiation genes.

### BRG1, ACTL6A and loricrin are downregulated in high-grade cSCCs

**Fig. 2:**
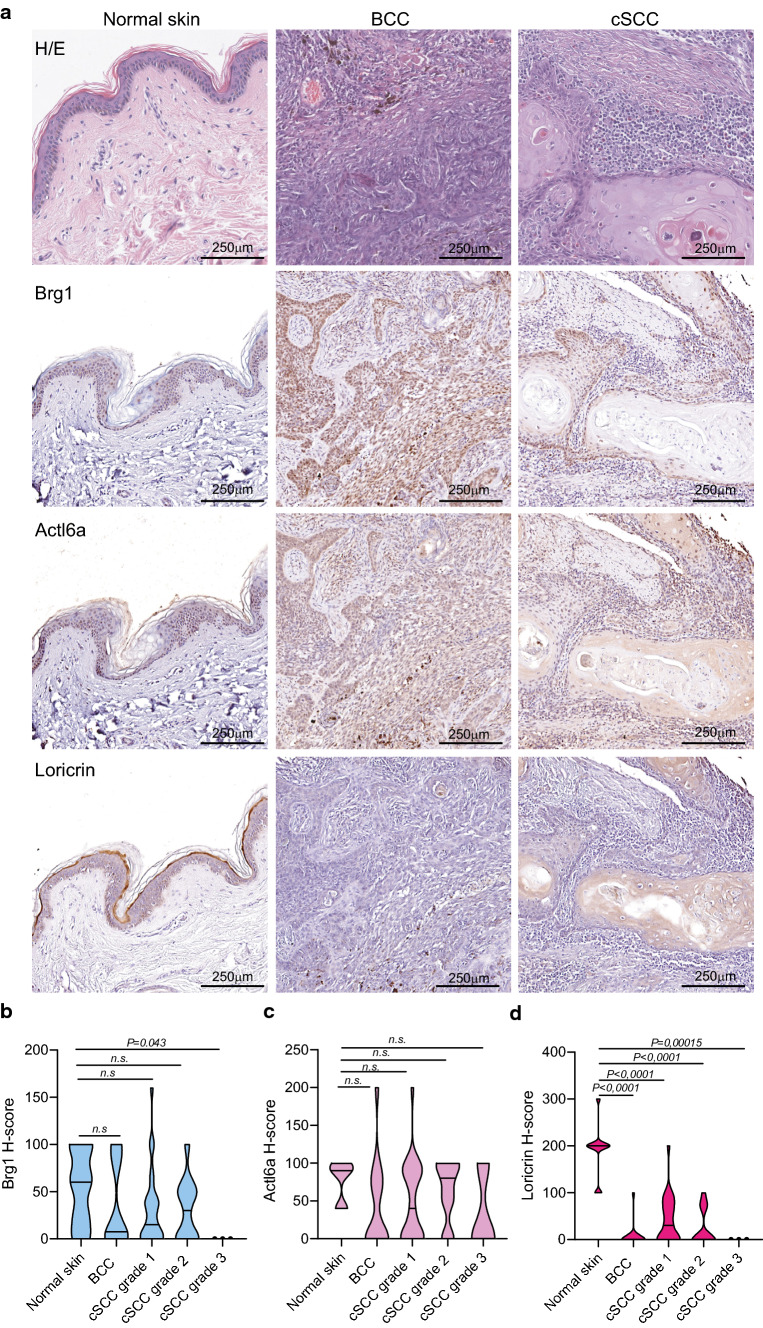
BRG1, ACTL6A and loricrin expression in BCC and high-grade cSCC. **a** H&E staining and IHC analysis of Actl6a, Brg1 and Loricrin expression in normal skin, cBCC and cSCC samples of different grades. Normal skin samples (n = 10), cBCC samples (n = 13), cSCC samples of grade 1 (n = 24), grade 2 (n = 13) and grade 3 (n = 3). **b** Histological-index (H-index) of Brg1. The p-value was obtained by Student’s T-test; *ns* not significant. **c** Histological-index (H-index) of Actl6a. The p-value was obtained by Student’s T-test; *ns* not significant. **d** Histological-index (H-index) of Loricrin. The p-value was obtained by Student's T-test; *ns* not significant

To further confirm the data obtained at the mRNA level for the patient cohort described in Supplementary Table S1, we analysed additional tumour samples using commercially available tissue microarray (TMA). Specifically, we analysed 10 samples of malignant melanoma, 39 samples of cutaneous squamous cell carcinoma of different grades and 13 samples of cutaneous basal cell carcinoma. Skin tumour samples were compared to 10 samples of normal skin (Fig. 2). As indicated by the H-score, expression of BRG1 and ACTL6A did not change significantly between normal skin and BCC (Fig. 2a–c), possibly due to the limited number of cases analysed. Instead, analysis of BRG1 expression in grade 1, grade 2 and grade 3 of cSCC samples confirmed decreased staining (p-value: 0.043) in poorly differentiated tumours compared to well-differentiated tumours and normal squamous epithelium (Fig. 2a–c). These data are consistent with the results obtained using the clinical samples previously analysed (Fig. 1) and suggested that the low expression of BRG1 and, as consequence the low SWI/SNF activity, correlate with poorly differentiated cSCC. In fact, loricrin downregulation was observed in grade 3 tumour samples (p-value: 0.00015)**,** suggesting that BRG1 might have a role in controlling the expression of EDC genes in cSCCs. Notably, loricrin was also downregulated in BCC and in grade 1–2 cSCCs (p-value: 0.001). This suggests that additional genetic and/or epigenetic mechanisms also contribute to tumour dedifferentiation. Interestingly, the expression patterns of BRG1/ACTL6A and loricrin were completely different in melanoma, another type of skin tumour derived from melanocytes (Supplementary Fig. 1), confirming a tumour-specific regulatory function of uc.291 and BRG1 in keratinocyte-derived malignancies.

### BRG1, BRM and ACTL6A expression in cSCC and squamous cell carcinoma datasets

To determine whether the obtained results are in line with additional cSCC data sets or in other type of squamous cell carcinomas, we examined GEO portal datasets for cSCC (GSE7553), oesophageal squamous cell carcinoma (oeSCC, GSE20347), and cervical squamous cell carcinoma (ceSCC, GSE7803). We analysed ACTL6A, BRG1, BRM and loricrin expression at the mRNA level in normal and in cancer tissues. We found a significant increase in ACTL6A expression in all cases (p-value: from 0.0252 to < 0.001). This observation parallels the genomic amplification described for ACTL6A in head and neck squamous cell carcinoma (HNSCC) [48] and confirms a possible function of ACTL6A as an oncogenic driver in squamous cell carcinomas of different origins. The expression levels of BRG1 and BRM, the two mutually exclusive catalytic subunits of the SWI/SNF chromatin remodelling complex, were unchanged and/or downregulated in different combinations. In cSCC (GSE7553) and ceSCC (GSE7803) datasets, BRM was downregulated (p-value: 0.0014 and 0.0015, respectively), with no significant change in BRG1. Expression of the loricrin gene was significantly downregulated only in ceSCC data set (p-value: < 0.001). Overall, these results confirmed that in most cases, BRG1/BRM expression is downregulated in squamous cell carcinomas of different origins, suggesting a role for the SWI/SNF chromatin remodelling complex in all carcinomas.

## Discussion

In normal epidermis, it has been shown that BRG1, the catalytic subunit of the SWI/SNF complex, binds to many regions within the keratinocytes EDC locus to control local remodelling of the chromatin fibre within the EDC at the nucleosome level in an ATP-dependent manner [49–52]. In contrast, the ACTL6A subunit modulates the SWI/SNF complex preventing its binding to promoters of EDC differentiation genes to maintain the undifferentiated status [53]. The transcript uc.291, by physically interacting with ACTL6A, releases the ACTL6A-mediated repression of differentiation genes, therefore, the SWI/SNF complex is able to interact with differentiation promoters and releases chromatin to allow the transcription of EDC genes [47]. The ACTL6A subunit is amplified and overexpressed in HNSCCs, playing a central role as oncogenic driver [48, 53]. This observation leads us to hypothesise a role for the transcript uc.291 and as consequence, for the SWI/SNF complex, in cSCCs. To date, very little is known on the contribution of long non-coding RNAs in squamous cell carcinomas malignancies in general and in non-melanoma skin cancers. Few non-coding RNAs have been associated to increased proliferation and migration capacities in cSCC (LINC00162, HOTAIR and LINC00520) [29, 37, 41–43]. However, the mechanism leading to cSCC de-differentiation observed in invasive poorly differentiated carcinomas has not been investigated, as well as the involvement of the SWI/SNF complex, responsible for chromatin releases to allow EDC genes transcription.

**Fig. 3:**
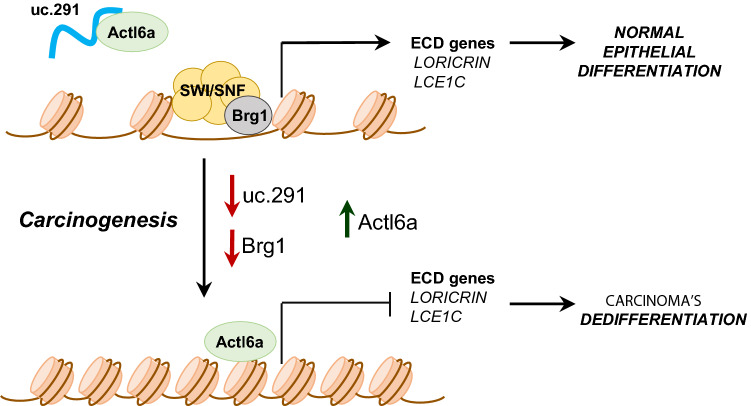
Schematic model of uc.29/ACTL6A/SWI/SNF complex action in cSCCs. The SWI/SNF complex (containing the Brg1 subunit) is important to allow the expression of EDC genes during keratinocytes differentiation. In cSCCs, down-regulation of uc.291 and Brg1 reduces SWI/SNF complex activity resulting in Actl6a binding in proximity to EDC genes and inhibition of loricrin and LEC1C expression

**Fig. 4:**
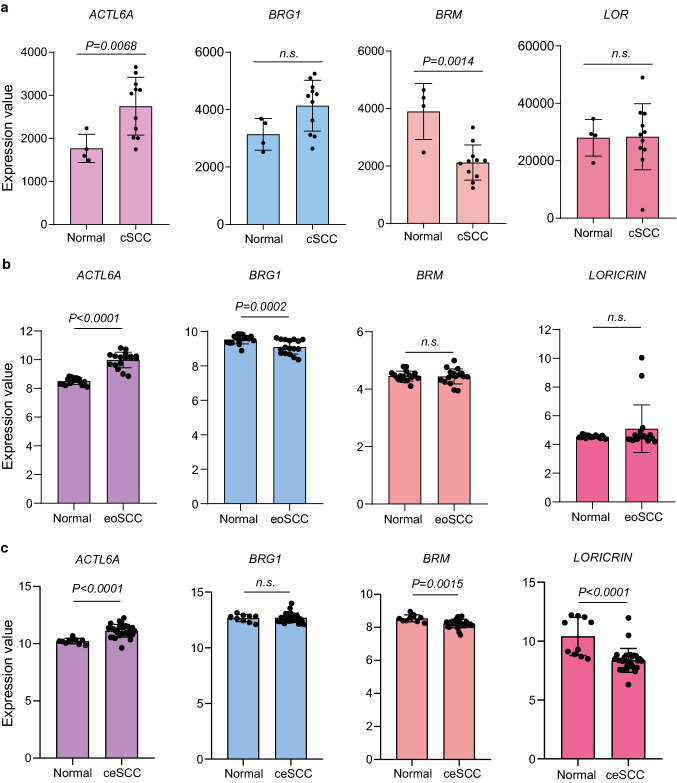
*ACTL6A*, *BRG1*, *BRM* and *LORICRIN* expression in cSCCs and in data sets of other types of squamous cell carcinoma. **a** Expression of *ACTL6A, BRG1, BRM* and their target *LORICRIN* in clinical samples of normal skin (n = 4) and cSCC (n = 11) using the GEO dataset GSE7553. The p-value was obtained by Student’s T test; *ns* not significant. **b** Expression of *ACTL6A, BRG1*, *BRM* and their target *LORICRIN* in clinical samples of oesophageal normal tissue (n = 17) and in oesophageal SCC clinical samples (n = 17) using the GEO dataset GSE20347. The p-value was obtained by unpaired Student’s T test; *ns* not significant. **c** Expression of *ACTL6A*, *BRG1*, *BRM* and their target *LORICRIN* in cervical normal tissue (n = 10) and in cervical SCC clinical samples (n = 28) using the GEO dataset GSE20347. The p-value was obtained by a unpaired Student’s T test; *ns* not significant

We observed a strong down-regulation by RTqPCR of uc.291 expression (Fig. 1) and detected ATCL6A expression in cSCCs (Fig. 2). Down-regulation of uc.291 reinforce the ACTL6A-mediated inhibition of the SWI/SNF complex activity in proximity of EDC promoters. Consequently, loricrin and LEC1C genes, located in the EDC locus, are strongly downregulated, both at mRNA and protein levels (Figs. 1, 2). These data, summarised in Fig. 3, indicated that the SWI/SNF complex (in particular the BRG1 subunit), together with the ACTL6A inhibitor uc.291, play an important role in the de-differentiation phenotype seen in poorly differentiated cSCCs. Interestingly, a similar pattern is also seen in BCC, when samples were analysed at mRNA level (Fig. 1), yet these results were not confirmed at protein level (Fig. 2) by TMA, suggesting that at least in BCC additional mechanisms are required to suppress the differentiation programme. Analysis of publicly available dataset (cSCC, oeSCC, ceSCC) confirmed the data described in cSCCs (Fig. 4), suggesting that down-regulation of uc.291 and BRG1/BRM are common features in SCCs of different origins. Altogether, these results expand the understanding of the epigenetic mechanisms accounting for tumorigenesis and identify novel tumour suppressors pathways in SCCs.

## Material and methods

### RNA extraction and real-time PCR analysis

Total RNA was isolated from clinical specimens using RNeasy Lipid Tissue Mini Kit (Qiagen, Hilden, Germany) and retrotranscribed by a SensiFAST cDNA Synthesis Kit (Bioline, Memphis, TN, USA) according to the manufacturer’s protocol. Real-time PCR was performed using GoTaq qPCR Master Mix (Promega, Madison, WI, USA). The primers used are listed in the Supplementary Table 3. Expression of each gene was defined by the threshold cycle (Ct), and relative expression levels were calculated by using the 2^−ΔΔCt^ method after normalization with reference to expression of TBP as a housekeeping gene.

### Immunohistochemical staining

A skin cancer tissue microarray containing 10 samples of normal skin, 10 samples of malignant melanoma, 39 samples of cutaneous squamous cell carcinoma, and 13 samples of cutaneous basal cell carcinoma was purchased from US Biomax (Cat. No. SK801c, Rockville, MD, USA). The samples analysed in Fig. 1 and listed in Supplementary Table 2 were collected at IDI-IRCCS hospital (CRI-NMSC) after approval of the ethical committee (Protocol No. 552/1, December 14, 2018) and prior patient consent. Immunohistochemical staining for ACTL6Aa and BRG1 was performed using an anti-ACTL6A antibody (Cat. No. 76682, Cell Signalling), anti-BRG1 antibody (Cat. No. 49360S, Cell Signalling) and anti-loricrin antibody (Cat. No. PRB-145P, Covance) following the manufacturer’s instructions [54]. For staining, sections were dewaxed and rehydrated and incubated to block endogenous peroxidases in a 0.03% solution of hydrogen peroxide in methanol. Antigen retrieval was then performed by boiling the sample in 0.01 M citrate buffer pH 6.0 for 15 min in a microwave. The slides were incubated with an anti-ACTL6A antibody (1:400), -BrRG1 antibody (1:150) or -loricrin antibody (1:1000) for 1 h at room temperature. Signals were detected using an UltraTek HRP anti-polyvalent DAB staining system (ScyTek, Logan, UT, USA), and the slides were then counterstained with haematoxylin, dehydrated, and mounted. The slides were scanned using a NanoZoomer scanner (Hamamatzu, Shizuoka, Japan) with a 40× objective. Images of haematoxylin/eosin staining for TMA were downloaded from the manufacturer’s website (www.biomax.us).

### Histological scoring of the samples

Samples were scored in a blinded manner by a pathologist using a semi-quantitative method. Cases were analysed for staining intensity, which was scored as 0 (not detected), 1+ (weak), 2+ (intermediate), and 3+ (strong). For each case, the histological “H-score” (0–300) was calculated by multiplying the percentage of positive cells (0–100%) by the intensity (0–3).

### Bioinformatic analysis

Normalized values for *ACTL6A*, *BRG1* and *LORICRIN* expression in skin cancer samples were obtained from the NCBI GEO portal. cSCC accession number: GSE7553; oeSCC accession number: GSE20347; ceSCC accession number: GSE7803.

### Statistical analysis

All statistical analyses were performed using GraphPad Prism 8.0 software (San Diego, CA, USA). For analysis of gene array data and protein levels from the tissue microarray experiment, the significance level (p) was calculated using Welch’s unequal variances t-test. Values of p < 0.05 were considered significant. Violin plots were generated in R using the ggplot2 package.

## Supplementary Information

Below is the link to the electronic supplementary material.

Supplementary file 1 (PDF 1174 KB)

## Data Availability

The authors declare that the data supporting the findings of this study are available within the paper and its supplementary information files.
