# Supplementary material for: Involvement of transcribed lncRNA uc.291 and SWI/SNF complex in cutaneous squamous cell carcinoma
Source: Discov Oncol. 2021 May 3;12:14. doi: 10.1007/s12672-021-00409-6 (PMC8777507; doi:10.1007/s12672-021-00409-6)
Supplement: Supplementary file 1 — (PDF 1174 KB) [file 12672_2021_409_MOESM1_ESM.pdf]

Supplementary table 1
Patients cohort analysed

VARIABLES		BCC	cSCC
**Age**			
	<70 years	7	2
	≥ 70	19	11
**Sex**			
	Female	6	7
	Male	20	6
**Tumour site**			
	Trunk	15	1
	Lower limb	2	2
	Upper limb	2	1
	Head and neck	7	9
**Tumour size (wide, cm)**			
	size ≤ 1	-	4
	1 < size <2	-	5
	size ≥2	-	4

BCC: basal cell carcinomas; cSCCs: cutaneous squamous cell carcinomas; -: not measured.

Supplementary table 2
Clinical and histological informations of TMA tissues specimens

TMA position	Age	Sex	Pathology diagnosis	Grade	*Brg1* H-score	*Actl6a* H-score	*Loricrin* H-score
A1	73	Male	Squamous cell carcinoma of right leg	1	5	70	20
A2	54	Male	Squamous cell carcinoma of chest wall	1	0	0	0
A3	67	Male	Squamous cell carcinoma of neck	1	0	0	0
A4	58	Male	Squamous cell carcinoma of right back	1	40	0	30
A5	90	Female	Squamous cell carcinoma of face	1	15	0	0
A6	84	Female	Squamous cell carcinoma of right face	1	0	0	0
A7	76	Male	Squamous cell carcinoma of right armpit	1	10	0	0
A8	73	Male	Squamous cell carcinoma of left leg	1	160	90	60
A9	74	Male	Squamous cell carcinoma of neck	1	15	5	0
A10	72	Male	Squamous cell carcinoma of left preauricula	1	40	10	80
B1	64	Male	Squamous cell carcinoma of right cheek	1	40	50	60
B2	79	Female	Squamous cell carcinoma of thigh	2	30	100	80
B3	56	Female	Squamous cell carcinoma of left lower limb	1	30	0	40
B4	34	Male	Squamous cell carcinoma of scalp	1	40	0	0
B6	61	Male	Squamous cell carcinoma of lip	1	0	100	80
B7	55	Male	Squamous cell carcinoma of left back of foot	1	60	100	90
B8	88	Female	Squamous cell carcinoma of right forehead	1	70	90	60
B9	65	Male	Squamous cell carcinoma of orbital cavity	1	100	90	200
B10	85	Female	Squamous cell carcinoma of right face	1	15	30	0
C1	80	Female	Squamous cell carcinoma of right face (sparse)	1	0	90	0
C2	37	Male	Squamous cell carcinoma of forehead	1	0	90	80
C3	56	Male	Squamous cell carcinoma of left lower limb	1	0	30	0
C4	66	Male	Squamous cell carcinoma of left back of foot	2	50	50	70
C5	55	Female	Squamous cell carcinoma of left heel	2	50	50	70
C6	66	Male	Squamous cell carcinoma of left preauricula	1	0	200	30
C7	67	Male	Squamous cell carcinoma of right shoulder	1	40	100	100
C9	60	Male	Squamous cell carcinoma of face	1	100	80	100
C10	69	Male	Squamous cell carcinoma of right fore shin	2	0	0	0
D1	47	Male	Squamous cell carcinoma of scalp	2	60	100	0
D2	64	Male	Squamous cell carcinoma post aurem	2	0	100	100
D3	51	Male	Squamous cell carcinoma of left thigh	2	50	60	0
D4	58	Male	Squamous cell carcinoma of lumbar part	2	0	0	0
D5	67	Male	Squamous cell carcinoma of left preauricula	2	40	80	0
D6	66	Female	Squamous cell carcinoma of nasal tip	2	20	80	0
D7	68	Male	Squamous cell carcinoma of occipitalia	2	0	100	0
D8	73	Male	Squamous cell carcinoma of chest	2	100	100	0
D9	71	Male	Squamous cell carcinoma of left thigh (sparse)	2	0	0	0
D10	45	Male	Squamous cell carcinoma of scalp	3	0	100	0
E1	72	Male	Squamous cell carcinoma	3	0	0	0
E2	70	Male	Squamous cell carcinoma of scalp	3	0	0	0
E3	64	Male	Basal cell carcinoma of scalp	-	5	0	0
E4	62	Male	Basal cell carcinoma of scalp	-	80	0	0
E5	57	Male	Basal cell carcinoma of scalp	-	0	0	0
E6	48	Male	Basal cell carcinoma	-	0	0	0
E7	74	Female	Basal cell carcinoma of right cheek	-	0	0	0
E8	74	Male	Basal cell carcinoma of lumbosacral area	-	100	100	0
E9	64	Female	Basal cell carcinoma of scalp	-	100	0	0
E10	48	Male	Basal cell carcinoma of submaxilla	-	0	0	0
F1	60	Male	Basal cell carcinoma of scalp	-	10	200	100
F2	54	Male	Basal cell carcinoma of left face	-	0	0	0
F3	38	Male	Basal cell carcinoma of right forehead	-	80	70	0
F4	63	Female	Basal cell carcinoma of right neck	-	20	80	0
F5	62	Female	Basal cell carcinoma of right face	-	100	80	10
F6	70	Female	Squamous cell carcinoma of scalp	-	100	50	0
F7	53	Male	Malignant melanoma of right sole of foot	-	100	200	200
F8	55	Male	Malignant melanoma of right forearm	-	70	0	0
F9	76	Male	Malignant melanoma of right medial malleolus	-	100	200	200
F10	78	Male	Malignant melanoma of left cheek	-	0	90	0
G1	65	Male	Malignant melanoma of right sole of foot (sparse)	-	100	200	10
G2	41	Female	Basal cell carcinoma of scalp	-	0	60	10
G3	74	Male	Malignant melanoma of left foot	-	20	30	0
G4	42	Male	Malignant melanoma of left heel	-	200	160	0
G5	60	Male	Malignant melanoma of right buttocks	-	0	100	0
G6	45	Male	Malignant melanoma of crissum (sparse)	-	0	40	0
G7	55	Male	Malignant melanoma of sole of foot	-	50	60	0
G8	52	Male	Malignant melanoma of right heel	-	0	100	0
H1	53	Female	Adjacent normal skin tissue of breast	-	0	40	200
H2	73	Male	Adjacent normal skin tissue of left heel	-	60	40	200
H3	75	Male	Adjacent normal skin tissue of left foot	-	100	90	200
H4	42	Female	Adjacent normal skin tissue of breast	-	70	90	300
H5	50	Female	Adjacent normal skin tissue of breast	-	60	90	200
H6	49	Female	Adjacent normal skin tissue of breast	-	100	40	200
H7	49	Female	Adjacent normal skin tissue of breast	-	0	100	200
H8	56	Male	Adjacent normal skin tissue of breast	-	30	100	200
H9	37	Female	Adjacent normal skin tissue of left chest wall	-	20	100	100
H10	62	Female	Adjacent normal skin tissue of breast	-	100	90	100

TABLE S2

Primers	Sequence 5'-3' direction
**TBP F**	CAAACCCAGAATTGTTCTCCTTATT
**TBP R**	GTCGTCTTCCTGAATCCCTTTAG
**ACTL6A F**	TTTTGGTGCAGAGCGGCTAA
**ACTL6A R**	ACACTGCCATAG
**BRG1 F**	AAAGGTTTGGAGTGGCTGGTGTC
**BRG1 R**	AGAGAGGCACGATGATGAGGAAGG
**UC291 F**	GCGTCAATGTTCATCTGTAATTC
**UC291 R**	CTGTTCTCAGCCTGTGCCGAG
**LORICRIN F**	CTCTGTCTGCGGCTACTCTG
**LORICRIN R**	CACGAGGTCTGAGTGACCTG
**LCE1C F**	GAATCCAGGACCGCAAACTG
**LCE1C R**	TGGACCTGTGAGCCTCTCAG

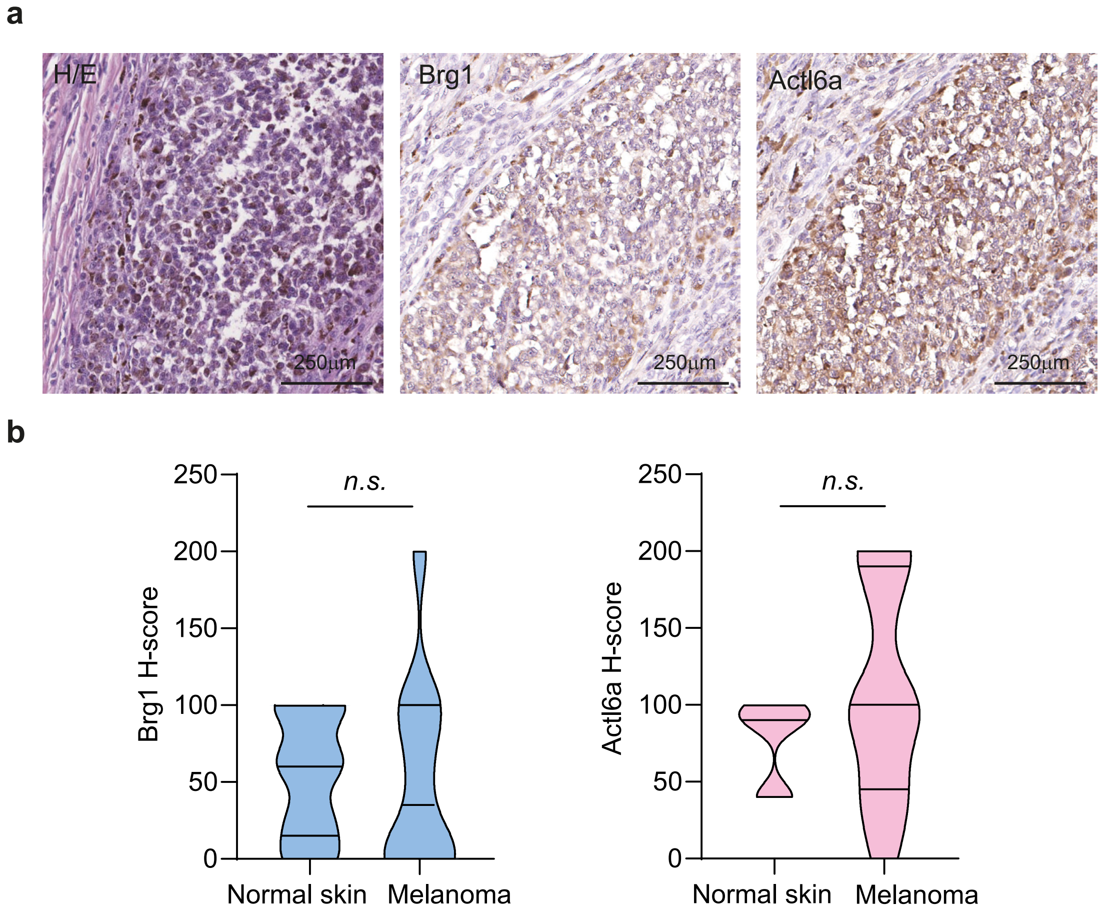

Supplementary Figure 1. Brg1 and Actl6a expression in melanoma. **a)** H&E staining and IHC analysis of Actl6a and Brg1 expression in melanoma (n=11). **b)** Histological-score (H-score) of Brg1 and Actl6a. The p-value was obtained by Student's T-test; ns= not significant.
